# Cellular Responses in Sea Fan Corals: Granular Amoebocytes React to Pathogen and Climate Stressors

**DOI:** 10.1371/journal.pone.0001811

**Published:** 2008-03-26

**Authors:** Laura D. Mydlarz, Sally F. Holthouse, Esther C. Peters, C. Drew Harvell

**Affiliations:** 1 Department of Biology, University of Texas at Arlington, Arlington, Texas, United States of America; 2 Department of Ecology and Evolutionary Biology, Cornell University, Ithaca, New York, United States of America; 3 Tetra Tech, Inc., Fairfax, Virginia, United States of America; University of Birmingham, United Kingdom

## Abstract

**Background:**

Climate warming is causing environmental change making both marine and terrestrial organisms, and even humans, more susceptible to emerging diseases. Coral reefs are among the most impacted ecosystems by climate stress, and immunity of corals, the most ancient of metazoans, is poorly known. Although coral mortality due to infectious diseases and temperature-related stress is on the rise, the immune effector mechanisms that contribute to the resistance of corals to such events remain elusive. In the Caribbean sea fan corals (Anthozoa, Alcyonacea: Gorgoniidae), the cell-based immune defenses are granular acidophilic amoebocytes, which are known to be involved in wound repair and histocompatibility.

**Methodology/Principal Findings:**

We demonstrate for the first time in corals that these cells are involved in the organismal response to pathogenic and temperature stress. In sea fans with both naturally occurring infections and experimental inoculations with the fungal pathogen *Aspergillus sydowii*, an inflammatory response, characterized by a massive increase of amoebocytes, was evident near infections. Melanosomes were detected in amoebocytes adjacent to protective melanin bands in infected sea fans; neither was present in uninfected fans. In naturally infected sea fans a concurrent increase in prophenoloxidase activity was detected in infected tissues with dense amoebocytes. Sea fans sampled in the field during the 2005 Caribbean Bleaching Event (a once-in-hundred-year climate event) responded to heat stress with a systemic increase in amoebocytes and amoebocyte densities were also increased by elevated temperature stress in lab experiments.

**Conclusions/Significance:**

The observed amoebocyte responses indicate that sea fan corals use cellular defenses to combat fungal infection and temperature stress. The ability to mount an inflammatory response may be a contributing factor that allowed the survival of even infected sea fan corals during a stressful climate event.

## Introduction

Emerging infectious diseases are triggered by a changing environment in many ecosystems and organisms, from humans to coral reefs [1]. In very few of our natural or managed ecosystems do we understand the immune mechanisms that influence disease resistance. Tropical corals are under unprecedented stress from pathogens and climate change [1]–[4]. Recently, the effects of these stressors on coral physiology have been highlighted [5]–[8] showing resilience of some corals to climate change and more physiological plasticity than previously expected. While such studies have examined coral heterotrophy [5], decalcification [6], symbiont exchange [7], and inducible secondary metabolites [8] as mechanisms by which corals can overcome changing ocean conditions, no studies to date have directly studied host immunity and plasticity of innate immunity in field conditions.

**Figure 1:**
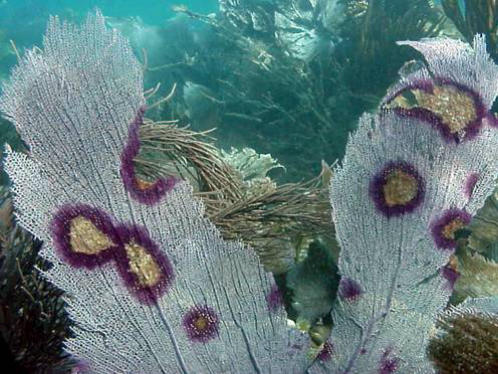
Picture of a sea fan coral (*Gorgonia ventalina*) infected with *Aspergillus sydowii*, multifocal purple annular lesions are indicative of infection (photo by Ernesto Weil).

The common sea fan *Gorgonia ventalina* is an exceptional model for cnidarian immunology and disease resistance since natural populations are experiencing a prolonged, widespread outbreak of a fungal disease caused by *Aspergillus sydowii*. This fungal-host pathosystem is easily identified in the field, easily manipulated and tractable in lab experiments [8], [9], [10]. Other species of the *Aspergillus* genera, such as *fumigatus* and *niger* are notorious as opportunistic pathogens of immune-compromised humans and insects [11] evoking mostly innate immune responses from their diverse hosts. Therefore studies with this pathosystem are not only relevant to marine ecology but to the evolution of cellular-based immune responses and the study of fungal diseases. Like its pathogenic counterparts, *Aspergillus sydowii* is hypothesized to also invade immune-compromised hosts [10], [11], [12] and causes deep tissue infections which manifest as darkened lesions (Figure 1). In these lesions, fungal hyphae have been observed in the coral axial skeleton alongside host-produced melanin, while an increase in pigmented calcium carbonate sclerites actually gives these lesions their distinct coloration [12]–[15]. Cellular components of immunity such as granular amoebocytes have been pictured circulating through the gelatinous matrix of the sea fan's mesoglea, the collagenous connective tissue linking the epithelial layers of epidermis and gastrodermis [10], [15], [16]. Amoebocytes have been shown to play a role in wound repair and tissue regeneration in sea anemones and similar gorgonian corals. In fact, the dramatic influx of these putative immunocytes to injured coral branches and subsequent phagocytic activity led this process to be defined as an inflammatory response [16], [17], [18]. In invertebrates the term inflammation is broadly used to define local reactivity of tissues following injury or infection in many taxa, including cnidarians [16]–[20].

The interplay of environment and innate immunity in basal invertebrates remains a black box even though such immunological studies could provide important tools to mitigate spread of infectious disease. Innate immunity of both vertebrates and invertebrates has been a recent focus, as it becomes increasingly clear that even vertebrates rely heavily on innate mechanisms that reach back evolutionarily to the most basal invertebrates [21], [22]. Studies of immune mechanisms, dynamics, and integration of environmental signals have focused heavily on model organisms, such as *Drosophila*, in controlled laboratory environments [23], with few studies examining natural populations exposed to a naturally changing environment. While temperature stress can enhance innate immunity and resistance to disease in many insects [24], the dramatic widespread die-offs of corals in response to thermal stress raise the question of how immunity interacts with temperature stress in cnidarians.

Temperature stress brought on by prolonged warming of sea water is the cause of coral death by bleaching and can facilitate some coral disease outbreaks [25], [26]. Uncertainty of the mechanism by which increased temperature leads to disease outbreaks has led to the hypothesis that temperature stress causes corals to be immune compromised, but there are no significant studies on this phenomenon to draw conclusions. In the Fall of 2005, the Caribbean experienced the largest warm temperature anomaly in more than 100 years [27], [28]; sea water temperatures were elevated for 4–8 continuous weeks in the Florida Keys and for 14 weeks in Puerto Rico. This temperature event caused mass bleaching and mortality of corals; in some cases 50% of live coral cover was lost [25], [27]. Following this mass bleaching, disease spread through the remaining population and caused increased mortality [25]. These combined events underscore the importance of elucidating the mechanism by which corals can fight temperature stress and infectious diseases concomitantly.

In this paper we characterize the local and systemic changes in amoebocyte density in sea fan tissue with emphasis on linking these cellular responses to natural events. We describe how sea fan tissue reacts to fungal infection with two of the mainstays of invertebrate immunology; melanization via the prophenoloxidase cascade and increased amoebocytes. Additionally we test the hypothesis that temperature stress affected immunity by measuring amoebocyte density using corals naturally heat stressed in the field during the 2005 Caribbean bleaching event as well as experimentally heat stressed. This data set combines field sampling of wild populations with experimentally obtained data, and is uniquely set against a backdrop of the longest and warmest temperature anomaly in the Caribbean which killed many reef-building corals.

## Results

### Melanization reaction and amoebocyte responses in natural infections

**Figure 2:**
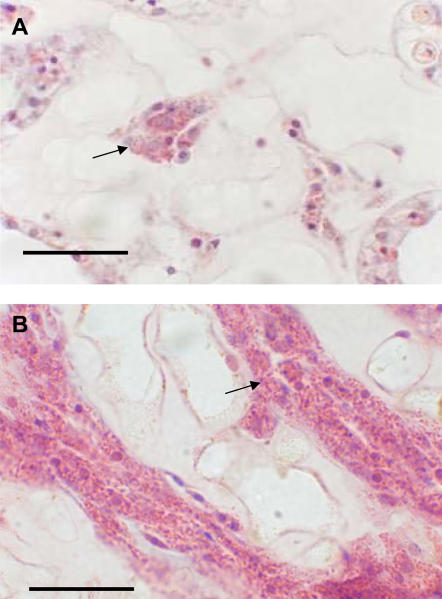
Amoebocytes in mesoglea (connective tissue) of naturally diseased sea fan corals. A) Healthy coral with granular amoebocytes dispersed in mesoglea as indicated by arrows. B) Diseased coral with an increase in granular amoebocytes in the mesoglea. Scale bar = 25 µm.

**Figure 3:**
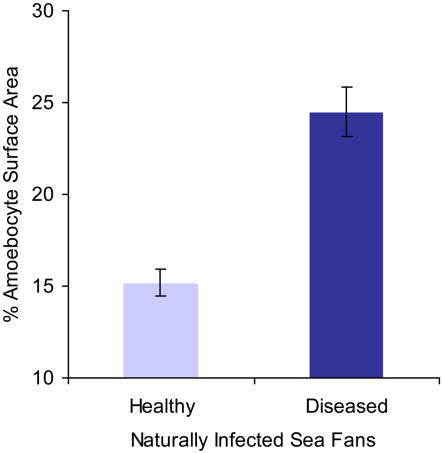
Quantitative analysis of amoebocytes in mesoglea of sea fan corals showing a dramatic increase in amoebocytes in diseased coral tissue. % surface area covered by amoebocytes calculated from histological images of mesogleal tissue sampled from uninfected sea fans and mesogleal tissue adjacent to fungal infections in diseased sea fans. Data presented are mean±s.e.m, n = 8 , *X* ^2^ 28.93, p<0.0001.

The cellular component of the sea fan's immune response to pathogen infection was quantified by calculating the area of mesoglea (tissue between the polyps) occupied by amoebocytes from histological images. We detected a sizeable and dramatic increase of the granular amoebocytes in tissue infected with fungus, indicative of a classic inflammatory response. In healthy corals the amoebocytes comprise an average of 15.2% of the mesogleal tissue area and appear uniformly interspersed in the tissue (Figure 2A). In diseased corals there are more amoebocytes aggregated in the mesoglea adjacent to areas with fungal infections and they occupy a greater surface area (24.5%) (Figure 2B, Figure 3, n = 8, *X* ^2^ = 28.93, p<0.0001).

**Figure 4:**
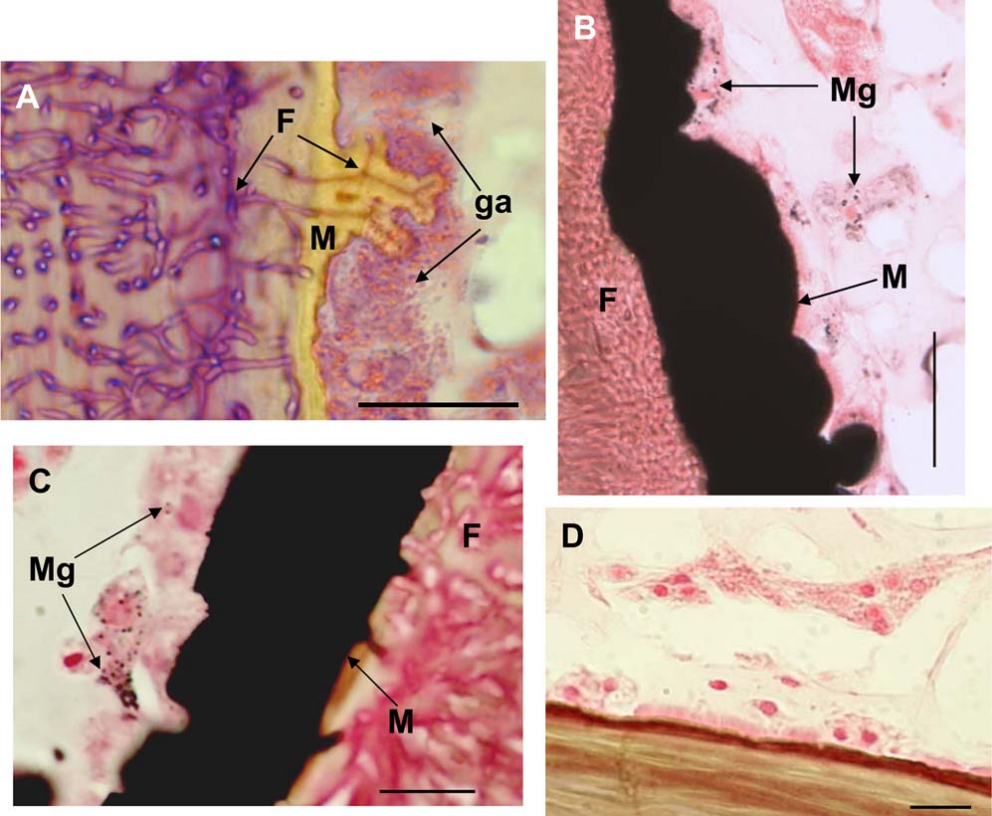
Histological images of sea fans infected with *Aspergillus sydowii* and presence of melanin both as a band and in amoebocytes as melanosomes. A) Histological preparation of an infected coral stained with H&E showing individual fungal hyphae (F) in skeleton and surrounded by melanin (M). Note granular amoebocytes (ga) aggregated near melanin and fungal hyphae. Scale bar = 25 µm. B) Histological preparation of an infected coral showing multiple amoebocytes containing melanin granules in contact with the thick layer of melanin preventing *A. sydowii* hyphae within the axial skeleton (at left) from contacting the sea fan tissue (on right). Fontana-Masson staining procedure, scale bar = 25 µm. C) Close up of an amoebocyte containing melanin granules (Mg) and black stained melanin (M) layer surrounding fungus (F). Fontana-Masson staining procedure, scale bar = 10 µm D) Uninfected coral stained with Fontana-Masson's procedure showing lack of melanin and melanin granules. Scale bar = 10 µm.

**Figure 5:**
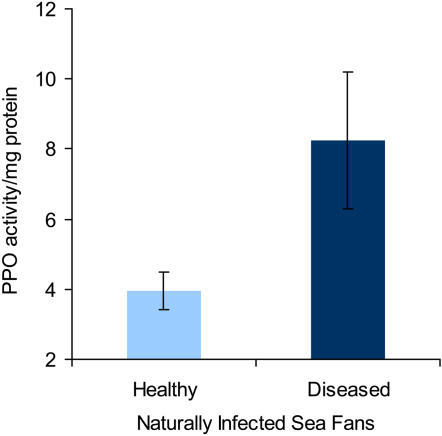
Prophenoloxidase activity in healthy and diseased sea fans as measured by the oxidation of L-dopa to dopachrome. Data presented are mean±s.e.m n = 12, F = 4.7, p = 0.040.

To determine the potential role of the amoebocytes in arresting infections and in melanin biosynthesis, we examined histological preparations of infected sea fans as well as activity of prophenoloxidase. In cross sections stained with hematoxylin and eosin (H&E) fungal hyphae can be seen infiltrating the proteinaceous gorgonin axis, and infected areas of axis are surrounded by layers of host-produced melanin as well as granular amoebocytes (Figure 4A). In addition, we used the Fontana-Masson staining procedure which causes melanin to appear black as a result of the reduction of silver nitrate to metallic silver [29], [30]. In Fontana–Masson stained sections, the layers of melanin surrounding the fungus in the skeleton appear black, furthermore, black granules (melanosome-like) can be seen in the granular amoebocytes (Figures 4B, 4C). No black precipitate is seen in non-infected controls (Figure 4D). Concomitant with melanin deposits and granular amoebocytes is a two-fold increase in prophenoloxidase activity as measured by oxidation of L-dopa to the colored dopachrome in infected tissue (Figure 5, n = 12, F = 4.7, p = 0.04).

### Amoebocyte response to experimental fungal exposure and spatial arrangement

**Figure 6:**
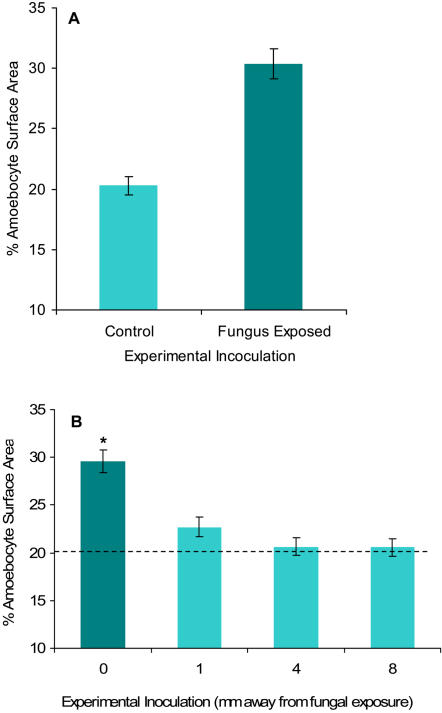
Quantitative analysis of amoebocytes in mesoglea of sea fans experimentally exposed to *Aspergillus sydowii*. A) Increase in amoebocytes in sea fans exposed to fungal hyphae, n = 14, *X* ^2^ = 41.85, p<0.0001. B) Fine spatial analysis of amoebocyte surface area at point of fungus exposure and 1, 4, and 8 mm away. Dashed line indicated control or basal % amoebocyte surface area. Data presented are mean±s.e.m, n = 8, F = 18.95, p = 0.015. Asterisk denotes significant differences at p<0.05.

**Figure 7:**
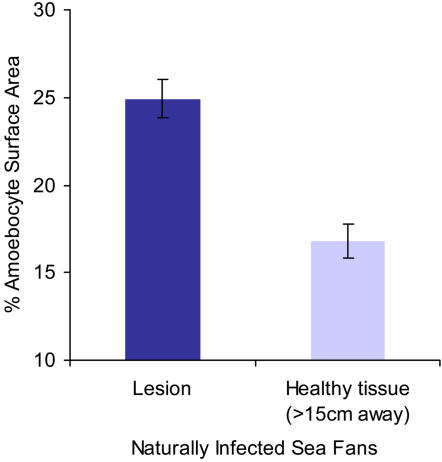
Amoebocytes are heterogeneously distributed within individual naturally infected sea fan colonies. Data presented are mean±s.e.m, n = 8, *X* ^2^ = 12.43, p = 0.0004.

To examine whether the increase in amoebocytes in infected tissue could be experimentally generated, we exposed corals to *Aspergillus sydowii* grown in culture and measured the amoebocyte response in histological preparations. Application of the fungus in wicks to the surface of the coral caused tissue necrosis and the formation of lesions with exposed coral skeleton that did not occur in sham-inoculated corals. Surrounding the areas of necrosis, amoebocyte surface area increased significantly above sham-inoculated corals. (Figure 6A, n = 14, *X* ^2^ = 41.85, p<0.0001). Fine spatial scale analysis revealed the increase was localized to the area immediately adjacent to the fungus; just 1 mm away from the lesion amoebocyte concentrations were the same as unaffected tissue (Figure 6B, n = 8, *X* ^2^ = 18.95, p = 0.0003). While the areas 4 and 8 mm away from the pathogen had fewer amoebocytes than the edge of the lesion, they did not have fewer amoebocytes than the basal levels in control corals, indicating that a simple local migration of amoebocytes to the affected area did not occur. On a larger scale, unaffected tissue (>15 cm away from the lesion) of naturally infected corals did not show an increase in amoebocyte numbers; further evidence against a systemic response (Figure 7, n = 8, *X* ^2^ = 12.43, p = 0.0004 ). Therefore, in response to *A. sydowii* invasion both by natural or experimental exposure, the coral amoebocytes are induced to increase locally closest to the areas of infection and pathogen penetration and there is no evidence for a systemic increase.

### Amoebocyte response to natural and experimental temperature stress

**Figure 8:**
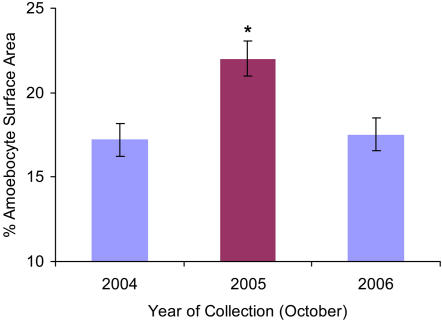
Amoebocyte surface area is higher in sea fans samples during the 2005 bleaching event. Data presented are mean±s.e.m, n = 6, *X* ^2^ = 24.51, p<0.0001. Asterisk denotes significant differences at p<0.05.

**Figure 9:**
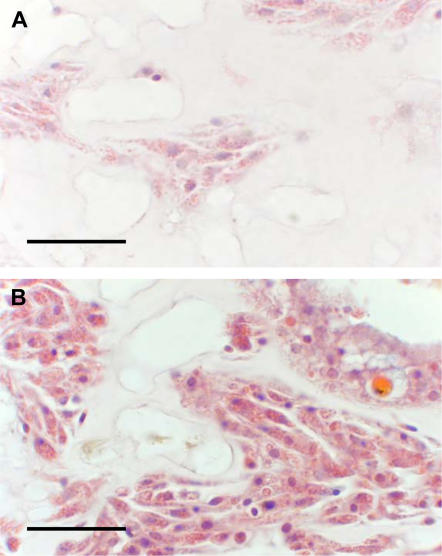
Amoebocytes in mesoglea (connective tissue) of sea fan corals exposed to experimental heat stress. Images are of the same coral colony, with A) one fragment kept at 29°C and B) fragment kept at 31.5°C. Scale bar = 25 µm.

**Figure 10:**
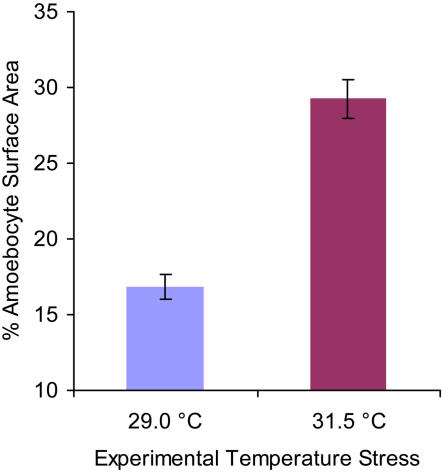
Increase in amoebocyte surface area in sea fans exposed to elevated temperature (31.5°C) for 8 days. Data presented are mean±s.e.m, n = 6, *X* ^2^ = 305.11, p<0.0001.

The average amoebocyte surface area in haphazardly chosen, healthy sea fans was higher during the temperature anomaly than during the year before and after (Figure 8, n = 6, *X* ^2^ = 24.51, p<0.0001) at the same marked reef site. In 2004 and 2006, the amoebocyte surface area comprised 17% of the coral tissue, approximately the same as in healthy, control sea fans (refer to Figure 5), indicating a stability of the natural unstressed coral amoebocyte populations. In 2005, at the height of the temperature anomaly, the corals had increased amoebocyte surface area to 22% of mesogleal tissue. To confirm temperature as a facilitator of increased amoebocytes, since this was the opposite of our prediction, we experimentally treated sea fan coral clonal replicates to increased water temperatures for 8 days. The increase in amoebocytes was evident in the histological images (Figure 9A and B) and quantified by image analysis (Figure 10, n = 6, *X* ^2^ = 305.11, p<0.0001.). The coral controls at ambient sea water temperature (27°C–29°C) averaged 16.9% amoebocyte surface area. However, the corals cultured at 31.5°C for 8 days increased their standing amoebocyte concentrations to 29.2%. This increase in amoebocytes was spatially homogenous and systemic within undamaged coral tissue. These data represent the first clear evidence of a systemic reaction to elevated temperature coordinated by the granular amoebocytes.

## Discussion

In this study we demonstrate the spatial dynamics of a localized inflammatory response of cnidarians to pathogens and a systemic inflammatory response to warm water temperatures. These stressors are two of the most destructive forces endangering coral reefs and understanding the underlying cellular mechanisms of resistance may open up new approaches to mitigation. This is the first demonstration that components of coral immunity play a role in both biotic and abiotic stressors, and at appropriate spatial scales.

In response to a biotic stressor, specifically a destructive fungal pathogen that resides in the deep tissue axial skeleton, there is an evident, localized increase in acidophilic granular amoebocytes in sea fan mesoglea. This combined with the presence of melanin (Figure 4A) points to local reactive changes in sea fan tissue consistent with an inflammatory response [17], [18], [19]. To further relate the melanin deposition and presence of amoebocytes, we used the Fontana-Masson staining procedure and identified melanosome-like structures in cells adjacent to melanin bands (Figure 4B,C). This may pinpoint the amoebocytes as sites of melanin synthesis and help to elucidate one of their functions. Not all the amoebocytes contained melanin granules, which may be a sign of the existence of subpopulations of amoebocytes each with their own characteristics or that amoebocytes can be activated to have different functions at discrete time scales [15], [17]. Concurrent with the presence of melanin and melanosomes, we measured an increase in prophenoloxidase activity in infected tissue. While the presence of melanin bands had been previously described in sea fan tissue, this is the first to identify melanin in the form of melanosomes and link the prophenoloxidase cascade to its synthesis, indicating that this important pathway in invertebrate immunity [31], [32] is present in sea fan corals as well.

In addition to halting fungal growth with cellular processes and a melanin physical barrier, the sea fan has been shown to produce lipid-based anti-fungal metabolites which can halt growth of *A. sydowii* in culture [33]. It has been demonstrated that the anti-fungal activity is higher in the purpled lesion than in non infected tissue [33], whether this activity is directly attributed to the amoebocytes or not, it is possible that these disease-fighting processes are all interconnected. There are likely additional functions of cnidarian amoebocytes, but based on this evidence and comparisons with other invertebrate immune cells [30], [31], [32], we hypothesize that the build up of amoebocytes is directly involved or facilitates deposition of melanin, and secretion of immune reactive enzymes and anti-fungal metabolites.

In addition to measuring an increase of amoebocytes in naturally infected sea fans, we were able to stimulate this response by experimentally exposing sea fans to fungus in culture. The amoebocyte aggregation within fungus-exposed sites was similar to the aggregation seen in natural infections (10.0% vs. 9.3% increase in naturally infected sea fans). Additionally, in both these experiments, there was spatial heterogeneity in the density of the amoebocytes, with areas adjacent to the fungal infections having the highest concentration of cells. The corals which we experimentally exposed to fungus lend themselves well to a finer-scale analysis of amoebocyte distribution in the mesoglea, since the point of original fungal contact was known. The areas bordering the fungal contact and amoebocyte aggregations had the same amoebocyte distribution as the controls in this experiment, but most importantly no fewer amoebocytes than the controls. This is an indication that cells were not simply migrating to the fungus-exposed areas from healthy areas. Such local migration was seen in an earlier study with the gorgonian coral *Plexaurella fusifera* [16], in which amoebocytes migrated to mesogleal tissue that was experimentally wounded. The tissue within 1 mm of the wounded edge had significantly more amoebocytes than the tissue around it, but the areas 2–4 mm away from the wounded edge had significantly fewer amoebocytes. It was concluded that the amoebocytes near the wounded tissue had migrated from other locations. In our study, we did not see this cell migration, so additional origins of the amoebocytes should be considered; amoebocytes may be proliferating at the site of infection or differentiating from a naive stem cell population often referred to as undifferentiated amoebocytes [16], [34]. Subsequent studies will be focused on elucidating these and related mechanism and are beyond the scope of this field-based study.

While the pathogen-stimulated inflammatory response in corals was the expected result, the same effect was not expected in reaction to elevated temperature. Nonetheless, an increase of amoebocytes was observed in sea fan corals naturally temperature stressed during the 2005 Caribbean-wide bleaching event, as well as experimentally temperature stressed sea fans in the lab. Because this response was not related to any visual signs of injury or infection on the sea fan, we conclude that it is a systemic response. Combined with the aforementioned potential roles of the amoebocytes in fighting pathogens and numerous existing studies that use numbers of circulating amoebocytes as a metric for immunocompetence [reviewed in 23] and [35], we hypothesize that this systemic response does confer (even if transiently) increased resistance. There are examples of both marine and terrestrial invertebrates where increases in immune reactive cells are correlated with increased air or water temperatures and improve disease resistance. In some insects, warmer temperatures activate greater immunity [24], specifically enhancing survival and perhaps cell-mediated encapsulation during host-parasite interaction [36]. Interestingly, some insects intrinsically thermoregulate to enhance cell-based immunity and encourage phagocytosis [37], [38]. In some bivalves and crustaceans [39], [40], [41], high levels of circulating hemocytes induced by warming were postulated to enhance immune capabilities in an attempt to protect against the high bacterial loads characteristic in summer waters. In marine ecosystems heterotrophic bacterial abundance is seasonally correlated to increased temperatures [42]; however, with the predicted warming of global oceans that will likely supersede these seasonal fluctuations an increase in bacterial growth and populations may occur [1], [4]. This may be more important in corals as they host a complex, rapidly changing bacterial community in their surface mucus layer [43]. In the sea fans, a priority is to examine whether temperature directly activates or indirectly induces amoebocyte density through an increase in surface bacteria. Whichever the proximate cue, it seems plausible that clades of cnidarians with this immune plasticity may be better suited for existing in a changing environment such as the projected warming.

Another component of the sea fan defenses that has been examined in the context of climate warming is the potency of the lipid-soluble, anti-fungal metabolites by Ward et al., 2007 [8]. They too demonstrated higher anti-fungal activity of sea fans during experimental heat stress and detected a dramatic (176%) increase in potency of anti-fungal metabolites extracted from the sea fans kept at 31.5°C (2.5°C above summer ambient) and exposed to fungus, relative to controls.

Taken together, these studies suggest an unexpected degree of resilience under adverse environmental conditions. Of course, it remains unknown how long the sea fan can maintain this high amoebocyte density in its tissues before it becomes energetically unfavorable or invokes self-harm to the host [31], [44]. However, the results from field sampling during the 2005 bleaching event are impressive in showing a persistent response even towards the end of the 3-month long heating event. Cellular cytotoxicity can be alleviated by induction of antioxidants, which have been shown to increase during warming in corals [45], [46]. At the very least, this potential for enhanced immunity could carry the sea fans through a period of time in which the conditions were unfavorable, as long as the stress was eventually alleviated and the physiological toll not too costly.

This type of resilience, in itself, may actually have ecological consequences as these prominent cellular and humoral components of sea fan immunity may give these alcyonacean corals an advantage over their scleractinian counterparts during unfavorable environmental conditions. Scleractinian coral tissue does not contain the high levels of amoebocytes seen in gorgonian coral tissue, in fact, the few small amoebocytes that can be detected in scleractinian corals are scattered throughout the mesoglea [10], [47], and induction of large aggregations of amoebocytes in diseased or bleached tissue has not been reported [47]. One priority in future studies will be to further examine the hypothesis that immune capabilities vary within the Cnidaria [17], such that gorgonian (octocoral) immunity is more robust in the face of pathogen attack and heat stress than scleractinian immunity (hexacoral). It is clear from the data presented in this paper that the sea fan aggressively combats infection in the gorgonian-*Aspergillus* pathosystem and exhibits the capability for resilience against multiple challenges.

## Materials and Methods

### Quantification of amoebocyte surface area

For histological analysis, sea fan samples were immediately placed in a fixative of 1 part buffered zinc-formalin concentrate (Z-fix™, Anatech, Battle Creek, MI) and 4 parts filtered sea water for at least 24 h and then decalcified in an aqueous solution of calcium citrate and formic acid. Histological samples were embedded in paraffin, sectioned at 4 µm, and stained with hematoxylin and eosin (H&E) or the Fontana-Masson silver stain protocol (ammoniacal silver nitrate stains melanin with nuclear fast red (Kernechtrot) counterstain) at the Cornell Veterinary School Histology Laboratory.

Each slide was divided into a grid comprised of 5-mm quadrats with a typical slide having 12–16 quadrats. Quadrats containing fungus were identified and randomly selected for analysis. In control and temperature-stressed sea fans grids were randomly chosen from all available quadrats on the slide. Within each selected quadrat, 3 replicate points were randomly chosen to photograph. Each coral slide was prepared from an individual sea fan colony and 6–14 sea fans were analyzed per treatment. For Figure 3 only, the tissue samples for analysis were an amalgam of sea fan samples collected in Akumal, Mexico, and Looe Key and Tennessee Reefs in the Florida Keys, in 2002, 2004, and 2005.

Photomicrographs were taken with a Nikon Coolpix 950 color camera in randomized quadrats under 100× magnification with an Olympus BH-2 compound microscope and transferred to *ImageJ* for analysis. A Hausser Scientific Company Bright Line Counting Chamber was used to calibrate measurements in each tissue image. The images were converted to black and white particles using the binary function and the threshold calibrated automatically. In *ImageJ* the threshold level is automatically determined by analyzing the histogram of the entire image. The threshold function divides the image into objects and background by taking a test threshold and computing the average of the pixels at or below the threshold and pixels above. For the images used in this study the threshold was automatically set at 1 for all analyses. Following threshold analysis, the particles were analyzed in the *ImageJ* program and the surface area of the darkly stained amoebocytes could be calculated within an image. At 100× magnification, an image contains only mesogleal tissue and the amoebocytes are the only cell type picked up by the threshold analysis. If other structures such as gorgonin, polyps, and solenia were less than 20% of the image after randomization, they were omitted from the image, if the random location landed completely on the gorgonin, solenia, and polyps an image of the mesoglea adjacent to the structure was taken. Total surface area of each image approximated 0.01 mm^2^, and amoebocyte coverage was expressed in percent of the total image surface area. Therefore, the data represent % amoebocyte coverage (as surface area) in the mesogleal tissue.

### Coral collection

Sea fan pieces were collected using SCUBA from the Looe Key Reef research site in the Florida Keys, USA. Specimens were collected from depths of 5–10 m. Sea fans showing signs of fungal infection such as lesion formation and purpled tissue were collected. Samples of healthy sea fans from the same reef area within 1 m distance were collected as controls. The samples were approximately 4 cm×4 cm in size and a 1-cm×2-cm subsection was cut for histological analysis. The presence of fungal hyphae in sea fan tissue was confirmed using histological preparations from the diseased lesions. For the yearly comparisons for the naturally heat-stressed corals, sea fans were randomly sampled from the same marked reef at the Looe Key Research Site in October 2004, 2005, and 2006. The October 2005 collection took place the second week of October, whereas this was the height of the bleaching event in the eastern Caribbean it was towards the end of the event in the Florida Keys. The Degree Heating Weeks, which measures the accumulation of thermal stress that coral reefs have experienced over the past 12 weeks, was between 7 and 9 on October 18^th^ 2005 and 1 on October 17^th^ 2006.

### Experimental exposure to fungus or elevated temperature

For experimentally manipulated corals, samples of healthy sea fans (n = 14, 9 cm×5 cm) were collected from Looe Key Reef research site in the Florida Keys, USA and brought to Mote Marine Laboratory's Tropical Research Laboratory (Summerland Key, FL). The fragments were sectioned into clonal replicates approximately 4 cm×3 cm in size and were kept in closed tanks in a flowing seawater table for a 2-day acclimatization period. Three replicate tanks were set up for each treatment. Fungal hyphae were grown in liquid PYG medium in sterile 1.5-ml epi-tubes from 1 ml of a 1×10^6^ spore/ml concentration of Florida Keys Isolate 1 (FK1) *Aspergillus sydowii*. Spores were incubated at 25°C for 1 week prior to experiment. At the time of inoculation the mass of fungal hyphae was removed from the medium and epi-tube using forceps and carefully applied to the surface of the sea fan. Fungal hyphae were kept in contact with the tissue by covering them with a gauze patch less than 1 cm^2^ in diameter threaded through the net of the sea fan using forceps. Controls received the same gauze patch without the fungus. The fungus and gauze patch remained on the sea fan for the entire duration of the experiment (8 days). Controls and fungal-exposed sea fans were harvested and the gauze patch removed, exposing a small lesion of necrotic tissue and exposed skeleton in the case of fungus exposure or unaffected tissue in the case of controls. For temperature-stressed sea fans the design was essentially the same, but sea fans did not receive fungal patches and were kept at ambient sea water which ranged from 27°C–29°C and in sea water warmed with aquarium heaters to 31.5°C for a period of 8 days. Three replicate tanks for each temperature were used. Temperature and salinity were measured daily and fresh or new seawater added as needed.

Histological samples from these experiments were obtained by immediately preserving the entire lesion with approximately 1-cm border in the Z-fix™ fixative. Histological preparations of sea fans exposed to *Aspergillus sydowii* did not exhibit signs of fungus growing in the gorgonin skeleton, as naturally infected samples do, but there were distinct signs of cellular rearrangement and amoebocyte aggregation, in addition to tissue necrosis and exposed skeleton. For the fine-scale analysis, 8 corals which demonstrated a definable lesion margin were chosen. The amoebocyte density at the margin was calculated. Then using a ruler on the slide, distances of 1 mm, 4 mm, and 8 mm were measured outward from the margin in a randomly predetermined direction using the grid made on the slide. Four directions on each coral slide were followed and 2 photomicrographs were taken at each distance to measure amoebocyte densities. For spatial analysis of amoebocytes in naturally infected sea fans (Figure 7), tissue adjacent (>15 cm) to the lesion that did not show signs of disease was collected during the October 2005 and July 2006 collection trips. Four sea fans from each of these trips were used in the analysis of diseased tissue and healthy tissue from the same sea fan.

### Prophenoloxidase Activity

Twelve healthy and twelve diseased sea fans were collected by SCUBA from Looe Key Reef research site in the Florida Keys, USA, during July 2006 and brought to Mote Marine Laboratory's Tropical Research Laboratory (Summerland Key, FL) where a 2 cm×2 cm piece was cut for histology and preserved in Z-fix™ (to confirm infection) and the remaining piece was flash frozen in liquid nitrogen. Frozen sea fan pieces were ground into a fine powder with a mortar and pestle then extracted in 0.2 M phosphate buffer, pH 7.8 with 5 mM 2-mercaptoethanol (Sigma-Aldrich, St. Louis MO) for 45 minutes on ice. The crude protein extracts were centrifuged at 405× *g*, the supernatants were recovered and centrifuged again at 14,000× *g* to remove more cellular debris. The protein concentration of each extract was determined using the Bio-Rad DC Protein Assay Kit (Hercules, CA) with bovine serum albumin as a standard. Extracts were stored at −80°C between assays.

Colorimetric measurements were calculated using a Synergy HT multi-Detection microplate reader with Gen5 software (Biotek Instruments, Winooski, VT). Prophenoloxidase is the inactive form of phenoloxidase, catalyzing the production of radical quinones, which are polymerized to melanin [32]. PPO activity was measured by diluting 10 µl of the extract in 50 µl sterile water (Sigma-Aldrich, St. Louis, MO) and monitoring the oxidation of L-DOPA (3-(3,4-dihydroxyphenyl)-L-alanine (Sigma-Aldrich, St. Louis, MO) to dopachrome. 25 µl of 10 mM stock of L-DOPA was added (2.2 mM final concentration) and the reaction was initiated by the addition of 20 µl of 50 µg/ml trypsin (Sigma-Aldrich, St. Louis, MO), which activates prophenoloxidase to phenoloxidase, and the absorbance was monitored at 490 nm for a period of 80 min. Data are presented as change in absorbance over 80 min/mg protein. Activity was completely inhibited by boiling the extracts for 10 min or by adding 50 mM sodium azide (data not shown).

### Statistical analysis

Due to the binomial proportion structure of the responses being analyzed, all data sets were power transformed using the Box Cox Y method in JMP Statistical Discovery Software version 5.1.2. (SAS Institute Inc., Cary, NC) to a scale in which they were normally distributed (Shapiro-Wilk). Variance testing (Levene) revealed that the variances were similar in the transformed scale. Histology data were analyzed using a mixed-effect linear model using PROC MIXED in SAS (SAS Institute Inc., Cary, NC), where the 4 quadrat measurements per coral slide were assumed to be equi-correlated. When multiple comparisons were made, the post-hoc Sheffe correction was applied to control the family-wise type I error probability. Prophenoloxidase data were analyzed using a one-way ANOVA in JMP Statistical Discovery Software version 5.1.2. (SAS Institute Inc., Cary, NC).
